# Emerging Role of Gallium-68 DOTANOC PET/CT Guided Radiofrequency Ablation in the Treatment of Tumor-induced Osteomalacia

**DOI:** 10.1210/jcemcr/luae044

**Published:** 2024-04-24

**Authors:** Jyoti Sharma, Rajeev Kasliwal, Tarun Jain, Gaurav Kant Sharma

**Affiliations:** Department of Endocrinology, Mahatma Gandhi Medical College and Hospital, Jaipur, Rajasthan, 302022, India; Department of Endocrinology, Mahatma Gandhi Medical College and Hospital, Jaipur, Rajasthan, 302022, India; Department of Nuclear Medicine, Mahatma Gandhi Medical College and Hospital, Jaipur, Rajasthan, 302022, India; Department of Interventional Radiology, Mahatma Gandhi Medical College and Hospital, Jaipur, Rajasthan, 302022, India

**Keywords:** tumor-induced osteomalacia, phosphaturic mesenchymal tumors, radiofrequency ablation, phosphaturia, gallium 68 DOTANOC PET/CT

## Abstract

Tumor-induced osteomalacia (TIO) is a rare acquired form of hypophosphatemia that can be cured when the tumor responsible is completely removed. These tumors can be small and located in anatomically challenging areas, rendering surgery both risky and extensive. Radiofrequency ablation (RFA) has been explored as an effective treatment option for such tumors. We present a case of a 35-year-old man exhibiting clinical and biochemical features consistent with TIO. The culprit lesion was not detectable on the whole-body computed tomography (CT) scan. Gallium (Ga-68) DOTANOC positron emission tomography (PET)/CT showed increased uptake in the left acetabulum and magnetic resonance imaging (MRI) confirmed the location of the tumor. Given the risky anatomical location, we opted for less-invasive RFA. Following an unsuccessful attempt at CT-guided RFA of the lesion, we used real-time Ga-68 DOTANOC PET/CT guidance for precise imaging during the ablation procedure. Our patient achieved complete remission both clinically and biochemically after RFA. This response was also evident by the absence of tracer uptake in follow-up imaging. In conclusion, DOTANOC PET/CT–guided RFA can serve as a safe and effective treatment for patients with tumors causing TIO. This modality proves valuable when surgical resection is not a viable option.

## Introduction

Tumor-induced osteomalacia (TIO) usually presents with complaints of bone pain, recurrent fractures at multiple sites, and whole-body weakness (1). TIO is caused by tumoral secretion of fibroblast growth factor (FGF)-23 (phosphatonin), which downregulates sodium-phosphate cotransporters (NaPi2a/NaPi-2c) and 25-hydroxyvitamin D 1-alpha-hydroxylase in the proximal renal tubules. This results in increased phosphate excretion and decreased intestinal phosphate absorption. FGF-23 also upregulates the expression of 25-hydroxyvitamin D 24-hydroxylase, a mitochondrial enzyme responsible for inactivating vitamin D metabolites (2). The biochemical hallmarks of TIO are hypophosphatemia, low tubular maximum reabsorption rate of phosphate to glomerular filtration rate (TmP/GFR), inappropriately normal or low 1,25-dihydroxyvitamin D, and normal or elevated plasma FGF-23 level.

Phosphaturic mesenchymal tumors (PMTs) are the most common tumor types associated with TIO (3). The localization of these tumors is challenging because they are very small and can be present virtually anywhere in the body. They are most commonly observed in the lower extremities, followed by the head and neck, torso, and upper extremities. Due to their expression of subtype 2 somatostatin receptors (SSTR2), the localization of these tumors has improved in the era of functional imaging (4, 5). Ga-68 DOTA SSTR PET/CT has better sensitivity than octreoscan and 18-fluoro-deoxyglucose (^18^FDG)-PET–CT in detecting culprit tumors in TIO and should be used as a first-line functional imaging technique. Functional imaging should encompass the entire body, including the arms and hands, which are routinely placed out of the field of imaging (5).

The standard treatment for TIO is surgical excision of the tumor (6). However, its presence in difficult locations, like deep in the bone or close to joints, makes surgical access and complete surgical removal difficult. Computed tomography (CT)-guided or ultrasound-guided RFA has been tried as an effective treatment option for tumors that are either visible on plain CT or ultrasound guided RFA (6, 7). We describe a case here of a phosphaturic mesenchymal tumor involving the left acetabulum whose culprit lesion was inconspicuous on plain CT and magnetic resonance imaging (MRI) but was visible on Ga-68 DOTANOC PET/CT.

## Case Presentation

A 35-year-old man presented with a history of widespread body pains and proximal muscle weakness lasting for 6 years. The pain initially began insidiously in the bilateral feet and subsequently progressed in severity, involving the knees, hip joints, trunk, and upper limbs. This progression ultimately confined him to bed for the past 4 months. Previous treatment for bone pain, including calcium, vitamin D, and analgesics, failed to provide any relief for his symptoms. There was no reported history of similar illness within his family.

## Diagnostic Assessment

**Figure 1.**
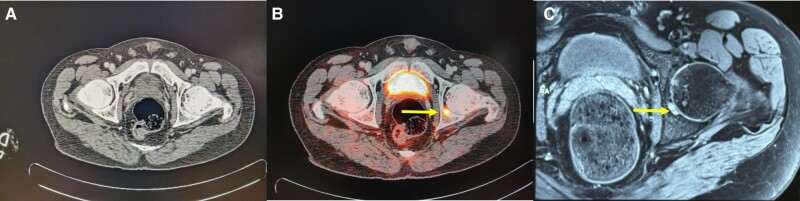
A, Plain computed tomography (CT) scan showing no lesion. B, Ga-68 DOTANOC PET/CT image showing tracer uptake involving posterior pillar of the left acetabulum with the lesion protruding intra-articularly. C, Magnetic resonance imaging scan showing a 3.5- ×1.5-mm lesion involving the posterior column of the left acetabulum and extending into the subchondral bone posterior to the fovea of the femoral head.

Biochemical investigations revealed hypophosphatemia, low TmP/GFR, and elevated FGF-23 levels, which are indicative of TIO (Table 1). A skeletal survey was performed and revealed multiple pseudofractures, suggesting the presence of metabolic bone disease. A whole-body CT scan failed to show a definite culprit lesion (Fig. 1A). Subsequently, the patient underwent Ga-68 DOTANOC PET/CT functional imaging, which showed a small area of tracer uptake located in the posterior aspect of the left acetabulum (Fig. 1B). To further investigate this finding, an MRI was performed, confirming the presence of an underlying tumor measuring 3.5- × 1.2-mm at the same site. The lesion extended into the subchondral bone posterior to the fovea of the femoral head (Fig. 1C). A multidisciplinary team consisting of an orthopedic surgeon and an interventional radiologist discussed the therapeutic options for the patient. It was felt that there was a high probability of surgical failure and out-of-proportion morbidity due to the small size and the deep location of the tumor. As an alternative treatment option, a single session of CT-guided RFA was offered to the patient.

**Table 1.** Laboratory values at baseline and follow-up

	Reference range and units	Baseline	1 wk after CT-guided RFA	3 mo after CT-guided RFA	1 wk after Gallium-68 DOTANOC–guided RFA	6 mo after Gallium-68 DOTANOC–guided RFA
Calcium	8.5-10.5 mg/dL (2.12-2.62 mmol/L)	8.9 mg/dL (2.22 mmol/L)	9 mg/dL (2.25 mmol/L)	8.9 mg/dL (2.22 mmol/L)	8.6 mg/dL (2.15 mmol/L)	8.7 mg/dL (2.17 mmol/L)
Phosphorus	2.6-4.5 mg/dL (.84-1.45 mmol/L)	1.8 mg/dL (0.58 mmol/L)	1.7 mg/dL (0.54 mmol/L)	2 mg/dL (0.65 mmol/L)	3.1 mg/dL (1 mmol/L)	4.2 mg/dL (1.36 mmol/L)
Alkaline phosphatase	44-147 U/L (733-2450 nkat/L)	1261 U/L (21 016 nkat/L)	1246 U/L (20 770 nkat/L)	1042 U/L (17 366 nkat/L)	380 U/L (6330 nkat/L)	134 U/L (2233 nkat/L)
25-hydroxyvitamin D	30-100 ng/mL (75-250 nmol/L)	32 ng/mL (80 nmol/L)		38 ng/mL (95 nmol/L)		41 ng/mL (102.5 nmol/L)
1,25-dihydroxyvitamin D	19.6-54.6 pg/mL (47-131 pmol/L)	20.1 pg/mL (48.24 pmol/L)		19.8 pg/mL (47.52 pmol/L)		34 pg/mL (81.6 pmol/L)
PTH	10-60 pg/mL (1.06-6.89 pmol/L)	84 pg/mL (8.9 pmol/L)		59 pg/mL (6.25 pmol/L)		62 pg/mL (6.57 pmol/L)
FGF-23	0-150 RU/mL	561 RU/mL		478 RU/mL	74.6 RU/mL	64.9 RU/mL
TmP/GFR	2.6-3.8 mg/dL (1.04-1.52 mmol/L)	1.4 mg/dL (0.56 mmol/L)		1.5 mg/dL (0.6 mmol/L)		3.3 mg/dL (1.12 mmol/L)
Creatinine	0.7-1.3 mg/dL (61.9-114.9 micromol/L)	0.67 mg/dL (59.24 micromol/L)		0.8 mg/dL (70.74 micromol/L)		0.62 mg/dL (54.82 micromol/L)

Abbreviations: CT, computed tomography; FGF, fibroblast growth factor; PTH, parathyroid hormone; RFA, radiofrequency ablation; TmP/GFR, tubular maximum reabsorption rate of phosphate to glomerular filtration rate.

## Treatment

**Figure 2.**
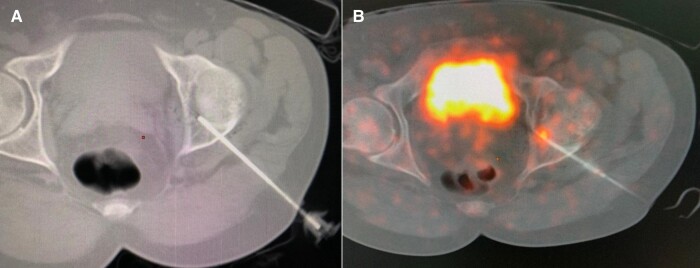
A, Computed tomography-guided radiofrequency ablation (RFA) needle in situ. B, Ga-68 DOTANOC PET/CT–guided RFA introducer needle tip placed at lesion tracer uptake site.

The procedure was thoroughly explained to the patient, and informed consent was obtained. The patient was kept in overnight fasting and sedation was administered during the procedure. To prevent cartilage damage during the ablation procedure, 15 mL of normal saline was injected into the left hip joint under ultrasound guidance. As the lesion was not visible on plain CT, we relied on the corresponding DOTANOC PET/CT–guided images to approximate the site. An insulated cannula was inserted under CT guidance, with the needle tip positioned at the approximate location of the tumor based on parameters derived from DOTANOC images. For the ablation, a RITA 1500 × RFA generator system with 8 prongs of multitipped electrodes (Side deployment starburst SDE multipronged electrosurgical device, Angiodynamics) was used. Heat ablation was performed at 90 °C with 60 watts of voltage for 5 minutes in each cycle (Fig. 2A). Subsequently, the needle tip was readjusted to the center of the intra-articular portion to cover the whole lesion with overlapping ablation zones. A total of 4 cycles were performed to achieve complete ablation of the lesion.

Post RFA, the patient underwent a 3-month follow-up period. His clinical condition remained unchanged throughout this period, with no alleviation of his symptoms. The biochemical profile revealed minimal improvement as indicated by FGF-23 and serum phosphorus levels (see Table 1).

After an unsuccessful CT-guided RFA session, a second session of ablation guided by DOTANOC PET/CT was contemplated following a consultation with the interventional radiology and nuclear medicine team. The patient was kept in 8 8-hour fasting state and sedated during the intervention. A total of 4 mCi Ga-68 DOTANOC was injected intravenously and after an approximately 45-minute uptake period, low-dose CT and PET images of the left hip joint were acquired with a Discovery IQ PET/CT scanner (GE Healthcare). No contrast was administered. Under real-time Ga-68 DOTANOC PET/CT guidance, an insulated cannula was inserted into the target area. The needle tip was positioned precisely at the level of tracer uptake, ensuring accurate targeting of the tumor site. A biopsy was taken from this site. We used electrodes similar to those employed in CT-guided ablation. Two cycles of ablation were performed using 60 watts of voltage, at 90 °C for 5 minutes in each cycle (Fig. 2B). The needle tip was readjusted and another 2 cycles using the same protocol were performed. A total of 4 cycles were performed with overlapping zones to achieve complete ablation of the lesion.

## Outcome and Follow-up

Following DOTANOC PET/CT–guided RFA, serum phosphorus, and FGF-23 levels normalized by the end of the first week (see Table 1). Histopathology was nonconfirmatory as tissue was insufficient.

Three months after the ablation procedure, marked clinical improvement with a significant decrease in pain and muscle weakness was noted. The patient regained the ability to sit up and demonstrated an overall increase in strength.

**Figure 3.**
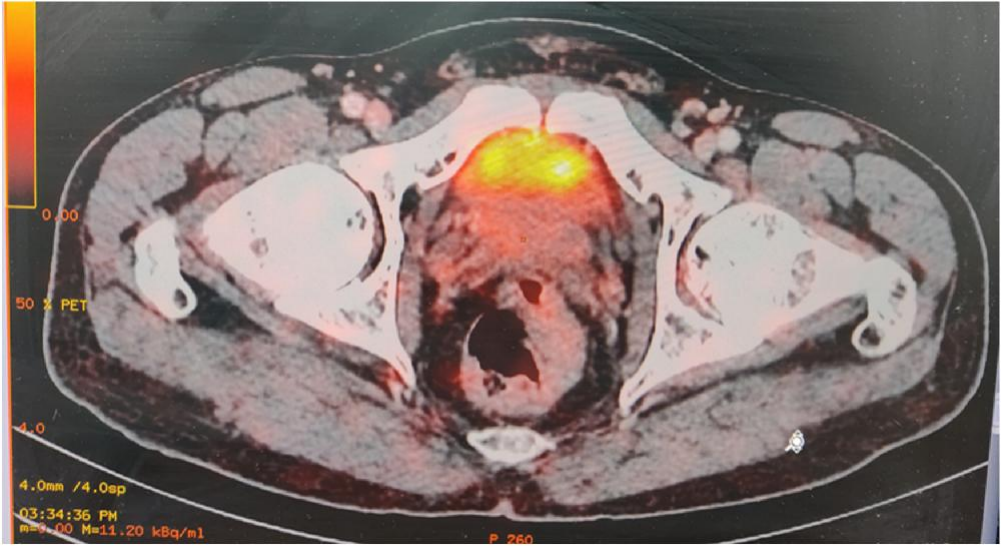
Post radiofrequency ablation, Ga 68 DOTANOC PET/CT scan showing the absence of uptake in the previously involved area.

At the 6-month follow-up post RFA, the patient was able to walk and resume routine activities without experiencing any pain or weakness. The follow-up MRI did not reveal any lesion at the previous site. Repeat DOTANOC PET/CT demonstrated a complete absence of tracer uptake, corroborating with clinical improvement (Fig. 3).

## Discussion

TIO is an uncommon paraneoplastic syndrome characterized by excessive phosphate wasting, leading to impaired mineralization of bone. The tumors causing this syndrome are variable in size and can be seen anywhere from head to toe (5, 8). In our study, the culprit lesion was localized in the posterior pillar of the left acetabulum.

In TIO, supplementation with phosphate and calcitriol is usually insufficient to alleviate symptoms, and the abnormal bone architecture tends to persist. The standard treatment is surgical excision of the mesenchymal tumor. However, the lesion is usually small, situated deep in the bone, and sometimes difficult to distinguish from the surrounding tissue. Complete excision requiring wide resection margins is necessary; the syndrome typically persists if any tumor tissue remains. This may cause iatrogenic tissue damage that is disproportional to the size of the tumor. In our case, the tumor was in a surgically inaccessible area, posing a challenge for complete surgical removal, and carrying the risk of considerable tissue damage.

There have been few case reports that have used RFA for treating patients with TIO in which the patients underwent ultrasound- or CT-guided RFA (6, 7, 9). DOTANOC (TOC or TATE) PET/CT has emerged as an ideal diagnostic tool for PMT compared to FDG PET/CT due to its superior detection rate (7). Maybody et al (10) have demonstrated the successful use of Ga-68 DOTATOC PET/CT for imaging guidance of a biopsy and cryoablation of a radiographically occult PMT. Our case further strengthens and highlights the effectiveness of Ga-68 DOTANOC PET/CT both for imaging guidance and ablation of inconspicuous PMT on anatomical imaging. RFA is a safe procedure with very rare complications (6, 7, 9, 10). Nerve injury, hematoma, infection, and cartilage injury are among the infrequent complications.

Burosumab (KRN23) is a fully human monoclonal antibody against FGF-23. Although it has been tried subcutaneously every 4 weeks in TIO in some parts of the world, it is currently unavailable in India. Also, opting for a definitive treatment would have been more cost-effective to our patient compared to lifelong burosumab (5).

A limitation of our report is that sufficient tissue was not obtained for biopsy to make a histopathological confirmation of the diagnosis. However, we demonstrated remission by clinical improvement, normalization of hypophosphatemia, and the disappearance of uptake on functional imaging. In the existing literature, including our patient, the follow-up data for patients who underwent RFA for TIO are limited to a maximum of 2 years. Long-term follow-up data are needed to compare the recurrence rates between RFA and surgical resection.

## Learning Points

- TIO should be included in the differential diagnosis in patients with progressive weakness, bone and muscle weakness, and multiple fractures in adults with hypophosphatemia.
- Although surgical resection is the standard of care, RFA can be a less invasive and safe modality of treatment in those patients for whom resection of the lesion is not possible because of inaccessible anatomical location or comorbidity that prohibits surgery.
- DOTANOC PET/CT–guided RFA is an effective treatment option for small functional tumors causing TIO that are inconspicuous on anatomical imaging and FDG PET/CT.

## Data Availability

Original data generated and analyzed during this study are included in this published article.

## Abbreviations

CT: computed tomography
FGF: fibroblast growth factor
Ga-68: gallium
MRI: magnetic resonance imaging
PET: positron emission tomography
PMT: phosphaturic mesenchymal tumor
PTH: parathyroid hormone
RFA: radiofrequency ablation
TIO: tumor-induced osteomalacia
TmP/GFR: tubular maximum reabsorption rate of phosphate to glomerular filtration rate
