# Correction to: “Emerging Role of Gallium-68 DOTANOC PET/CT Guided Radiofrequency Ablation in the Treatment of Tumor-induced Osteomalacia”

**DOI:** 10.1210/jcemcr/luae111

**Published:** 2024-05-31

In the above-named article by Sharma J, Kasliwal R, Jain T, and Sharma GK (*JCEM Case Reports*. 2024, 2(5); doi: 10.1210/jcemcr/luae044), there was an error in the Affiliation list.

In the originally published article, the affiliations for Tarun Jain and Gaurav Kant Sharma read: “^1^Department of Endocrinology, Mahatma Gandhi Medical College and Hospital, Jaipur, Rajasthan, 302022, India.” In the corrected article, Tarun Jain is affiliated with the “Department of Nuclear Medicine, Mahatma Gandhi Medical College and Hospital, Jaipur, Rajasthan, 302022, India” and Gaurav Kant Sharma is affiliated with the “Department of Interventional Radiology, Mahatma Gandhi Medical College and Hospital, Jaipur, Rajasthan, 302022, India.”

The author and affiliation list originally appeared as:

Jyoti Sharma,^1^ Rajeev Kasliwal,^1^ Tarun Jain,^1^ and Gaurav Kant Sharma^1^

^1^Department of Endocrinology, Mahatma Gandhi Medical College and Hospital, Jaipur, Rajasthan, 302022, India

The corrected author and affiliation list appears as:

Jyoti Sharma,^1^ Rajeev Kasliwal,^1^ Tarun Jain,^2^ and Gaurav Kant Sharma^3^

^1^Department of Endocrinology, Mahatma Gandhi Medical College and Hospital, Jaipur, Rajasthan, 302022, India

^2^Department of Nuclear Medicine, Mahatma Gandhi Medical College and Hospital, Jaipur, Rajasthan, 302022, India

^3^Department of Interventional Radiology, Mahatma Gandhi Medical College and Hospital, Jaipur, Rajasthan, 302022, India

The article has been corrected online.

doi: 10.1210/jcemcr/luae044

